# Geneticizing Ethnicity and Diet: Anti-doping Science and Its Social Impact in the Age of Post-genomics

**DOI:** 10.3389/fgene.2017.00056

**Published:** 2017-05-09

**Authors:** Jaehwan Hyun

**Affiliations:** Interdisciplinary Program in History and Philosophy of Science, College of Natural Sciences, Seoul National UniversitySeoul, South Korea

**Keywords:** human enhancement, anti-doping science, sports doping, geneticization, genomics and race, regulatory science

## Abstract

While gene doping and other technological means of sport enhancement have become a topic of ethical debate, a major outcome from genomic research in sports is often linked to the regulation of doping. In particular, researchers within the field of anti-doping science, a regulatory science that aims to develop scientific solutions for regulating doped athletes, have conducted genomic research on anabolic-androgenic steroids. Genomic knowledge on anabolic-androgenic steroids, a knowledge base that has been produced to improve doping regulation, has caused the ‘geneticization’ of cultural objects such as ethnic identities and dietary habits. Through examining how anti-doping genomic knowledge and its media representation unnecessarily reify cultural objects in terms of genomics, I argue that Ethical, Legal, and Social Implications (ELSI) research programs in human enhancement should include the social impacts of anti-doping science in their discussions. Furthermore, this article will propose that ELSI scholars begin their academic analysis on anti-doping science by engaging with the recent ELSI scholarship on genomics and race and consider the regulatory and political natures of anti-doping research.

## Introduction

While the use of science for doping detection in sports has long been a hotspot of ethical debate, an increasing number of social and ethical concerns have been raised by new genomic knowledge in relation to sport enhancements. Recent developments in genetics and genomics create new technological possibilities for enhancing sports performance on the molecular level. In particular, with the rise of gene therapy since the early 2000s, scientists have begun to worry about the misuse of gene therapy for sport enhancement (Baoutina, 2011). Since then, the World Anti-Doping Agency (WADA), the international organization coordinating and monitoring the illegal use of drugs in sports, has proactively implemented preventative strategies on gene doping; for example, in 2003, WADA added gene doping to the list of banned practices (WADA, 2009). Along these lines, bioethicists have discussed the social and ethical impacts of genetic modification in sports performance in doping practices (Miah, 2004; Sandel, 2009).

Ironically, major outcomes of genomic research in relation to doping in sports tend to be linked to doping regulation rather than to doping itself. In particular, since 2003 new genomic knowledge on anabolic androgenic steroids (AAS) has vastly increased because WADA has encouraged scientific researchers to find new doping detection methods for preventing gene doping and other unknown techniques (Miah, 2011). Anti-doping scientists, who aim to develop scientific and technological solutions for regulating doped athletes and who are mainly funded by international and national anti-doping agencies, have conducted genomic research on AAS. The scientific outcomes of their research play an important role in controlling international and national sports contests, because their findings are immediately introduced into doping regulation practice through WADA and national anti-doping agencies. Furthermore, the genomic knowledge they have produced has strongly influence popular thinking on sports performance, nutrition, and other topics, via news media on international matches like the Olympic Games and FIFA World Cup. Despite the importance of anti-doping science, few discussions of the social and ethical implications of this science exist (Hyun, 2016).

In this article, I argue that Ethical, Legal, and Social Implications (ELSI) research programs in human enhancement should consider the social impact of anti-doping science, particularly in relation to genomics. This paper examines the case of genomic research on the relationship between a uridine glucuronosyl transferase 2B17 (*UGT2B17*) gene and testosterone metabolism. By doing that, this paper shows how genomic research originally produced for strengthening doping regulations and the media’s hype on the implication of their research have caused the reification of cultural objects—ethnic identities and diet customs—in terms of genomics. This reification has led elite athletes and the lay public to understand their cultural differences as genetic differences. Due to this unnecessary reification, some athletes may face unjustified accusations of doping.

To investigate the social impact of anti-doping science in relation to genomics, this article conducts two analyses: content analysis of scientific journals and content analysis of news media. I used various sources to search for scientific articles and news reports related to anti-doping genomic studies. My first analysis is a content analysis of anti-doping genomic research. In order to collect this data, I first sought genomic research projects that received research funds from WADA between 2001 and 2016. This literature review of WADA-funded genomic research led me to recognize the association research between the *UGT2B17* and testosterone metabolism as a main research subject in anti-doping science. For this reason, I chose anti-doping research on *UGT2B17* as a case study of content analysis. I then collected scientific articles related to this subject on Google Scholar, using the terms “*UGT2B17*,” “testosterone,” “doping,” and “genomic research.” I also conducted a supplementary search to find *UGT2B17* research that might not have been identified by Google Scholar, specifically identify scientific reports on *UGT2B17* and testosterone metabolism that were uploaded as web resources on 34 WADA-accredited anti-doping laboratories.

My second analysis is a content analysis of media reports on anti-doping genomic research. Since many of scientific reports on *UGT2B17* and testosterone metabolism highlighted the genetic specificity of East Asians in relation to *UGT2B17*, I included news articles published in East Asian countries in my analysis. As a result, I examined four languages (English, Chinese, Japanese, and Korean) papers that reported anti-doping scientists’ *UGT2B17* research from January 1, 2001 to December 31, 2016. I used news meta-search engines to identify these articles: Google News (UK and US news articles), Baidu News (Chinese news articles), Yahoo Japan News (Japanese news articles), and Naver News (Korean news articles).

This article consists of three sections. In the first section, I introduce the current status of genomic research in relation to doping regulations. In the next section, I describe two “geneticization” cases with respect to genomic knowledge of *UGT2B17* and testosterone metabolism. Then, in the discussion section, I propose that the social impact of anti-doping science should be seriously considered in ELSI programs in human enhancement and genomics. Finally, through engaging with recent ELSI studies on genomics and race, I investigate the ways in which ELSI scholars may begin meaningful analysis of anti-doping science.

## Historical Background: Doping Regulation and Genomic Research

Ever since the International Olympic Committee (IOC) established its Medical Commission in 1967 and installed the Subcommision on Biochemistry and Doping in Sport in 1980, the field of scientific research for doping regulation has grown rapidly. One of the most important charges for doping regulation was to develop biochemical tests to detect AAS. As early as 1971, the IOC president Avery Brundage had asked the IOC Medical Commission for a method of detecting AAS (Dimeo, 2007, p. 112). Pioneering anti-doping scientists, like British pharmacologist Dr. Arnold Beckett at the University of London and German biochemist Dr. Manfred Donike at the German Sport University Cologne, sought to find an AAS screening method under the sponsorship of the IOC during the 1970s. The outcome of their biochemical and pharmacological research on AAS was the invention of a basic testing method: the testosterone/epitestosterone (T/E) ratio test. A major rationale of this test was that administrating exogenous testosterone does not affect the concentration of urinary epitestosterone glucuronide; if the ratio of testosterone glucuronide to epitestosterone glucuronide in urine is high, it should indicate the injection of exogenous testosterone. In 1982, the IOC introduced the T/E ratio test to deter AAS doping and set a T/E ratio in excess of 6.0:1 as a criterion for evidence of the injection of exogenous testosterone (Krieger, 2016).

Discovering detection methods of AAS continued into the 2000s. This effort to develop detection methods was partially due to the limitation of the T/E ratio test, given that test results were sometimes inconclusive. For example, doped athletes could avoid detection by taking low-dose AAS or suspending the use of AAS before the test. WADA, the new international anti-doping foundation established in 1999, tightened the doping test by adopting use of isotopic ratio mass spectrometry for AAS detection, in which an urinary T/E ratio of greater than or equal to 4.0 was considered indicative of doping; however, this new rule did not fully resolve the problem (Saudan et al., 2006).

Anti-doping scientists began to start genomic research on doping in sports by raising concerns about the limitations of the T/E ratio test. Since most anti-doping scientists were mainly experts in the fields of biochemistry, clinical chemistry, and pharmacology, their genomic research was naturally related to pharmacogenomics. They conducted pharmacogenomic studies of AAS that sought to understand AAS metabolism in the body with respect to environmental and genetic influences, and to find alternative detection methods.

In 2001, for instance, Drs. Anders Rane and Mats Garle at the Department of Laboratory Medicine, Division of Clinical Pharmacology, Karolinska Institute began a research project titled “Human Androgen Metabolism, Kinetics and Excretion: Genetic and Ethnic Determinants of Variation,” under financial support from WADA. In their research proposal, they proposed that the T/E ratio test “is probably affected by inter-individual and ethnic genetic differences and variation” and planned to identify polymorphisms in androgen metabolizing enzyme genes (Rane and Garle, 2001). Over the course of a decade, their group discovered that polymorphisms in several enzyme genes, like *UGT2B17*, cytochromes P17 (*CYP17*), and phosphodiesterase 7B (*PDE7B*), substantially influence the results of the T/E ratio test (Rane and Ekström, 2012). They contended personalized surveillance strategies in doping tests were needed to account for genetics-related individual differences in the T/E ratio test; for example, longitudinal monitoring of the T/E ratio of individual athletes would provide better results than a single T/E ratio test for all participants (Rane and Garle, 2001). Their proposal on individual surveillance strategies in doping tests supported WADA’s new anti-doping programs such as the Athlete Biological Passport program (ABP), which collects a longitudinal record of biological markers in individual athletes and detects doping violations when the recent biomarker results show large changes from the previous records.

In sum, the beginning of anti-doping genomic research was simultaneous to changes in AAS doping management practices. Given the limitations of the traditional T/E ratio test, anti-doping authorities and scientists began to seek alternative ways of regulating athletes. They developed more personalized surveillance on the biological status of each athlete; pharmacogenomic research highlighting genetic variability in testosterone metabolism contributed to introducing AAS to doping detection practices. No one regarded the social implications of genomic research with respect to doping in sport. Yet as we will see, the outcome of their research would deeply influence the way athletes thought about their profession.

## Geneticizing Cultures and Biological Determinism

Twenty-five years ago, sociologist Dr. Abby Lippman coined the term “geneticization,” defining it as “an ongoing process by which differences between individuals are reduced to their DNA codes, with most disorders, behaviors and physiological variations defined, at least in part, as genetic in origin” (Lippman, 1991, p. 19). Lippman’s idea is quite old but remains useful in describing how the scientific results of genomic research with respect to doping in sports, with its media representations, reify cultural differences as genetic differences. Though genomic scientists reject the conception of a gene as a blueprint, the ways that anti-doping scientists have adopted of speaking on the scientific results of genomic research have contributed to the development of genetic determinism on cultural activities related to sports. Media reports on their research, in turn, strengthen the gene-deterministic picture on cultural activities.

In this section, I describe how anti-doping scientists and media representations on their research reify ethnicity and diet as genetic beings, through the lens of geneticization. This rough sketch will provide the basis for further discussions on the social impacts of anti-doping science.

### The Geneticization of Ethnicity

As I showed in the previous section, anti-doping scientists have conducted pharmacogenomic research on the metabolism of AAS since the early 2000s. In particular, they have been interested in the effect of genetic variations in the metabolism of AAS in the body and the resulting T/E ratio test. By so doing, they wanted to show the existence of intra-individual differences in the T/E ratio and to problematize traditional doping test methods. For example, the Karolinska research group found that a deletion polymorphism in *UGT2B17* prevents encoding of the UGT enzyme to catalyze the glucuronidation of testosterone. They reported that research subjects who have this deletion polymorphism (del/del) in *UGT2B17* only excreted a small amount of testosterone glucuronide in their urine and had a T/E ratio test that was lower than threshold, despite an injection of AAS (Schulze et al., 2008). In addition, they discovered that the T > C polymorphism of the *CYP17* gene is related to urinary glucuronide levels of epitestosterone and ultimately affects the results of the T/E ratio test. Lastly, a genome-wide association study found that research subjects who were homozygotes of the G-allele in the *PDE7B* gene and who were injected with AAS had a lower T/E ratio than others who had an A-allele in *PDE7B* gene (Ekström et al., 2011).

*UGT2B17* is a fascinating genetic marker precisely because of AAS regulation research. It has been an important research topic for many anti-doping scientists around the world, including researchers at the Swiss Laboratory for Doping Analyses (hereafter, SLDA). Dr. Pierre-Edouard Sottas, the director of SLDA, designed experiments on the effect of *UGT2B17* that were similar to the Karolinska group’s experiments, whereby soccer players were screened for binary polymorphisms of *UGT2B17* (Strahm et al., 2009). The SLDA and Karolinska groups were convinced that they had discovered scientific grounds to problematize the traditional T/E ratio test and introduce a new doping practice—ABP. Indeed, Sottas pointed to the outcome of the *UGT2B17* study as scientific proof of the limitation of the T/E ratio test and later took the position of ABP Manager at WADA.

The study of *UGT2B17* contributed to the introduction of new regulation programs like ABP into doping detection practices. The problem, however, is that anti-doping scientists designed their experiments to employ the category of “ethnicity” as a variable used to identify genetic differences among research subjects. In 1999, the United States Institute of Medicine recommended that the nation’s National Institutes of Health focus on “ethnic groups” rather than “racial groups” in their cancer surveillance program. In this recommendation, the Institute of Medicine defined racial groups as groups related by biological commonalities and ethnic groups as groups related by cultural and behavioral commonalities (Oppenheimer, 2001). According to this definition, anti-doping scientists should have classified their research subjects into several cultural or social groups who did not *necessarily* have biological commonality. In fact, human geneticists have employed the category of ethnicity when labeling populations in terms of similarities and differences in common cultures (Panofsky and Bliss, 2017, p. 64).

In research designations, however, anti-doping scientists instead revitalized racial classifications that connote biological ties among people by employing the category of ethnicity. For example, Schulze et al. (2008) categorized their research subjects—Swedes and Koreans—into Caucasians and Asians. Furthermore, the Karolinska group was convinced that the deletion polymorphism of *UGT2B17* is “common in East Asians but relatively rare in Caucasians” (Schulze et al., 2009, p. 368). The SLDA group also adopted “ethnic origin” as a variable used to identify the metabolic effects of the *UGT2B17* genotype among research subjects: they reported that the distribution of the *UGT2B17* deletion polymorphism and T/E ratio threshold varied among “African, Caucasian, Asian, and Hispanic” participants and contended that Asians’ genetic characteristics allowed them to pass the T/E ratio test by lowering their T/E ratio threshold to below 4:1 (Strahm et al., 2009). Based on these studies, Karolinska and SLDA researchers have concluded that “ethnicity,” as “an endogenous factor,” plays a significant role in “connection to androgen metabolism” in the evaluation of an individual steroid profiling (Schulze et al., 2009; Rane and Ekström, 2012; Kuuranne et al., 2014). In consequence, they made ethnicity not a cultural status, but a genetic one. According to their works, ethnicity determines athletes’ androgen metabolism and thus allows specific ethnic groups to avoid the T/E ratio test genetically.

The media reports worsened this misuse of group categories, by which they misrepresented the outcome of *UGT2B17* studies as being implied to racist statements. *Reuters* reported that “steroid doping tests currently used … ignore vital ethnic differences in hormone activity.” The rapporteur stated, “individuals with a deletion of certain genetic “letters” on this [*UGT2B17*] gene —notably Asian men— excrete less testosterone in their urine” (Hirschler, 2009). Sports writer David Epstein wrote that genetic variations of *UGT2B17* benefit some athletes “to dope with impunity” in *The Sports Gene*. Epstein stated, “Two-thirds of Koreans have the genes that confer immunity to T/E ratio testing,” whereas only “about 10% of people with European ancestry have” (Epstein, 2013, p. 148). Nick Harris, sports news writer of *Mail on Sunday* criticized Asian athletes who are “born to cheat.” Harris exaggerated the implication of *UGT2B17* studies by saying that “a landmark Swedish study [of the Karolinska group] found that ‘doping with impunity’ gene variant [—the *UGT2B17* deletion polymorphism—] occurs in 66.7% of Asian populations and almost 10% of Caucasians” (Harris, 2013). Harris defined Asian athletes as those who “have a license to dope” and contended, “certainly official WADA statistics show that certain major accredited labs in some Asian countries are returning many fewer negatives than counterparts elsewhere” (Harris, 2013). By so doing, Harris suggested that readers consider Asians to be innately dishonest.

The way in which anti-doping scientists conflated “ethnicity” with “race” in their genomic research allowed the media to make racist arguments about sports doping. Byrd and Hughey (2015) claim that genetic determinism—race is genetically inherited—is one of the ideological double helix that shapes beliefs about racial inequality. This case demonstrates their argument well. A cultural category (ethnicity) became a biological category (race) in anti-doping research on *UGT2B17*; ethnic groups became understood as ones who shared a genetic inheritance; finally, this categorical change was utilized to make racist arguments in the popular media.

### The Possible Future of Epigeneticizing Diet

Although the case of ethnicity in *UGT2B17* studies and its media reports appears to be a typical example of geneticization and genetic determinism, the case of dietary habits and doping that I will examine in this subsection seems to be an atypical example, because of its epigenetic characteristics. In fact, epigenetic research has been considered a main opponent of genetic determinism. Recent sociologists of science, however, suggest that the epigenetic framework can be compatible with genetically deterministic views and some epigenetic explanations in both scientific practices and popular journals are narrated as deterministic (Waggoner and Uller, 2015). A similar trend is found in genomic research on doping and nutrition. This new current of anti-doping research aims to find the epigenetic influence of dietary habits in doping practices. By focusing on the role of environment in changing genetic regulatory mechanisms, the genomic research on doping and nutrition seems to steer anti-doping authorities and athletes away from the tendency to view genetic traits as deterministic of doping outcomes. This subsection will show how these new trends of research and their media representations may instead strengthen genetically deterministic views on doping and nutrition, rather than liberating people from genetic determinism.

In 2012, Declan P. Naughton and colleagues at the UK’s Kingston University reported that dietary green tea may lower the T/E ratio by suppressing testosterone glucuronidation. Under the sponsorship of WADA, they discovered that the catechin compounds included in green tea inhibit the enzyme UGT2B17 and thus may affect the relative ratio of testosterone glucuronide in urine (Jenkinson et al., 2012). This endocrinal effect of catechins on the human body was very inconclusive because it was merely an *in vitro* study.

The outcome of this study immediately invoked a strong reaction from anti-doping officials and scientists, however. At the beginning of the 2012 London Olympics, WADA officials, such as the scientific director Olivier Rabin, interpreted the result as a sign to introduce ABP into doping regulation practice in lieu of the traditional T/E ratio test. Also, a well-known anti-doping scientist, Charles Yesalis at Pennsylvania State University, expressed a deep concern that, “there are already lots of athletes out there drinking loads of green tea” to avoid doping detection (Cheng, 2012). Further, anti-doping scientists who took part in ABP programs quickly picked up dietary green teas as “exogenous factors,” influencing steroid profiling in relation to AAS detection (Kuuranne et al., 2014).

As with the case of the role of ethnic differences of the *UGT2B17* genetic polymorphism and its impact on doping tests, the mass media hyped and misrepresented the biological effect of drinking green teas on AAS screening. *The Guardian* ran a news report titled, “Green tea could hide testosterone.” *Daily Mail* also delivered this news using the title, “Green tea could help cheats;” *USA Today* had a similar title, “Green tea could cloud Olympic doping tests” (Associated Press, 2012; Cheng, 2012; Sportsmail Reporter, 2012). These sensationalist news title lines underplayed the point that Jenkinson et al. (2012) was just a preliminary *in vitro* study.

In fact, anti-doping studies that showed a remarkably different results from Jenkinson et al. (2012)’s study were completely ignored by WADA and the mass media. In 2013, the Karolinska group reported that the *in vivo* testing on non-steroidal anti-inflammatory drugs in 23 healthy males showed those drugs had no influence on the T/E ratio in urine. Based on this result, they argued that speculation on the inhibitory effect of drinks like green tea that were only based on *in vitro* studies should be seriously reconsidered (Lundmark et al., 2013, p. 6). Neither news reports nor WADA announcements accompanied this negative finding of dietary effects of green tea on real doping practice.

Meanwhile, the epigenetic effects of green tea catechins have simultaneously been investigated in relation to the rise of nutritional epigenetics among anti-doping scientists since that time. Nutritional epigenetics hypothesizes that food is a key environmental factor in altering the genetic regulatory mechanism of the human body (Landecker, 2011). Green tea intake is hypothesized to help prevent several tumors; epigallocatechin gallate, a type of catechin, is thought to block methylation of tumor-promoting genes (Yiannakopoulou, 2015). Though most studies on the epigenetic influence on nutrition have focused on carcinogenesis, in recent years anti-doping scientists have begun to focus their attention to the role of nutrition—including catechin components—on DNA methylation and its implications on doping practice (Schwarzenbach, 2011). In this context, the concept of epigenetic doping testing is proposed to identify behavioral and environmental factors that influence both epigenetic profiling and doping test outcomes (Diel et al., 2015; Andrèn-Sandberg, 2016, p. 4379). Under this new scientific vision on doping and nutrition, dietary activities—like drinking green tea—become an epigenetic action that influences the genetic regulatory mechanism of the human body.

Meloni and Testa (2014) anticipate that the epigenetic vision of life will reorder social norms as well as living phenomena. According to them, epigenetic findings will blur the distinction between natural and social inequalities through revealing the fact that societal factors such as class inequalities can modify biological endowments. With blurring boundaries between natural and social inequalities between human populations, the epigenetic vision will move to redefine cultural and social subpopulations as biological groups that can be identified with epigenetic markers. Furthermore, this epigenetic reordering of social populations will require new social norms for these populations. A specific subpopulation is expected to have an advantageous or disadvantageous genetic regulatory mechanism due to an epigenetic effect on their social and cultural activities; they might be restricted in their activities by social policy and discourse. For example, the US government’s fish consumption regulations based on studies on epigenetic effect of methylmercury exposure much more focus on controlling the dietary habits of Native Americans (Mansfield, 2012).

This possibility of epigeneticizing cultural and social groupings and making new social norms through epigenetic facts have already been seen in the case of drinking green tea and doping practices. Indeed, different consumption patterns of dietary green teas among different social groups affect the lives of individuals along cultural lines; some individuals who maintain drinking teas as a part of their lifestyle begin to worry about the *biological* implications of their *cultural* activity. Although the biological effect of green tea is not limited to specific cultural groups, the social and political context of sports doping forces particular ethnic groups to be more anxious about its biological implications than other ethnic groups.

For several years, a strong concern about tea drinking customs has been raised in the sports communities of East Asian countries such as China, Japan, and South Korea. Green teas are daily necessities in these countries; drinking tea, which is sometimes called a tea ceremony (*chayi* in Chinese, *chado* in Japanese, and *dado* in Korean), is one of the most distinctive East Asian cultural customs. Yet, due to criticism of green tea drinking as a potential crime because it can mask illegal uses of AAS, athletes and coaches in East Asian countries have suppressed their own cultural customs. In fact, a UK news media outlet implicitly connected Chinese athletes with drinking green tea when reporting the news of tea intake as a way to hide doping: “Chinese Gold medal winners at the Beijing games were given this [green] tea as a special present to recognize their achievements at the Beijing Olympics. But Olympic doping officials are now faced with the conundrum that this green tea may be used as a way of masking elevated levels of testosterone” (AP Television, 2012).

While English-written news reports did not consider about the possibility of incautious misuse of green tea in order to avoid doping detection among athletes in their own nations, East Asian newspapers dealt with the possibility very seriously. The Chinese news journal *Fenghuang Web* delivered the news by saying, “*Qing Ming Jie*, which falls on April 4–6, is the time to deliver new tea products into the [Chinese] market; yet who could imagine that neat, elegant green teas have been an umbrella for hiding AAS?” (Chen, 2012a). Other Chinese journalists also wrote: “Experts say that green tea works as an umbrella to hide doping and that [2012] London Olympic medalists shall be reexamined,” “athletes hope to drink green tea to hide their doping,” or “green tea became the subject for anti-doping monitoring because a scientific study discovered that this tea contains banned substances” (Chen M., 2012; Chen, 2012a,b). In Japan and South Korea, news writers reported this news in a tone similar to that of their Chinese colleagues. Their news reports made East Asian athletes very anxious. Chinese sports athletes were curious about whether green tea is a doping substance or not. In the Online Q&A Forum of the Korean Anti-doping Agency, one can easily find Korean athletes who continuously ask about whether drinking green tea and functional products containing green tea is considered a doping activity or not (Korean Anti-Doping Agency [KADA], 2016). In Japan, a Japanese drinking company Kirin’s new tea “*Harecha*,” which contained green teas and geranium, provoked controversy among Japanese athletes who suspected that this new product contained banned substances (Netorabo, 2015).

With the rise of epigenetic doping testing and the growing interest in epigenetic studies on doping and nutrition among anti-doping scientists, one can anticipate that more and more athletes will develop an epigenetic determinism of doping and nutrition—a belief that a specific diet pattern alters genetic regulatory mechanisms to help hide doped experiences. It is plausible that a popular racist discourse connoting epigenetic determinism might appear, due to epigenetic findings on doping and nutrition and due to the media’s misrepresentation on these findings.

Millard Baker, who is the founder of MESO-Rx.com, one of the largest AAS information websites, provides an example of how racist arguments can appear in relation to anti-doping genomic research. In a web article titled, “Green Tea Helps Steroid-Using Athletes Beat Anti-doping Test,” Baker contended that, in 2008, he realized that “genetically gifted” athletes, mostly “Asians,” have a doping advantage through the genetic effect of the *UGT2B17* deletion polymorphism. He wrote that “an athlete’s ethnicity may give them a doping advantage,” citing statistics on the percentage of *UGT2B17* deletion polymorphism among different human groups like “66.7% East Asian,” “29.1% Black,” “3.5% White Caucasian.” His diction of “genetically gifted” is not value-neutral, because it implied that Asians have “genetically unfair traits.” Since then, he had suspected the existence of a pharmaceutical drug blocking the UGT2B17 enzyme. Discovery of green tea’s endocrinal effect on the UGT2B17 enzyme in 2012 convinced him that drinking green tea as a method to avoid detection was “common knowledge in some elite athletes” (Baker, 2012).

Although Baker did not explicitly define who “some elite athletes” are, one can easily suppose that he tried to connect drinking green tea as a method for hiding doping with *genetically gifted Asian* athletes, through use of *UGT2B17* as a connector. The rise of epigenetic determinism due to anti-doping research is still in the future, yet it is genuinely possible.

## Discussion: Analyzing the Social Impacts of Anti-Doping Science

Sports doping has received extensive academic attention in the ethical literature on human enhancement (Tolleneer et al., 2012). The use of biomedical interventions to improve the physical performance of athletes in sports has served a paradigm case to ethical discussions on unnaturalness and unfairness in human enhancement.^1^ In this context, bioethicists and sport sociologists struggle with whether doping is unfair and unnatural or not, and try to create a role for new genetics in doping by only focusing on the unnaturalness of an unfulfilled enhancement technology—gene doping (Miah, 2004; Murray, 2012). For them, anti-doping science is a timid, minor subject. Furthermore, bioethicists who think performance enhancements are unnatural as well as unfair contend that anti-doping science should be pursued (Murray, 2012). On the contrary, critical sport sociologists who suspect that ethical values such as fairness and naturalness are social constructs believe that anti-doping science is just a scientific instrument to control and monitor the behaviors of sport athletes around the world (Park, 2005). Both sides do not consider anti-doping science a part of the social, ethical discussion in relation to human enhancement.

However, ELSI scholars in sport enhancements and genomics needs to pay attention to the rapid growth of regulatory research against sports enhancement over two decades, particularly in terms of genomics. Anti-doping scientists preemptively developed regulatory knowledge and technologies for expected doping technologies. Under WADA’s official systematic support, genomic research for regulation developed prior to the advent of a new gene doping. What’s more, anti-doping science has considerable power to change popular beliefs on the human body, sports, and nutrition through global sports contests such as the Olympic Games and FIFA World Cup. The scientific results of anti-doping science are more widely shared among the public—particularly athletes and coaches—than other academic sciences because of its global influence through international and national sports games.

In this paper, I described the social impact of anti-doping science in relation to its recent genomic research on AAS. Two cases of geneticization—ethnicity and dietary habits—show anti-doping science’s influential power on society, particularly in relation to thoughts concerning human enhancement. It indicates that ELSI scholars need to include this science in their analysis if they want to thoroughly investigate which ethical and societal problems will be raised with respect to the topic of sports enhancements and genomic science.

In this section, I analyze the social and ethical problems of anti-doping science in further detail. To do this, I engage with the ELSI literature regarding genomics and race. This detailed analysis provides insight into how the social impact of anti-doping science can be investigated as a part of ELSI research.

The ELSI scholarship exploring the topic of genomics and race shows that research practices and media representations cause biological reification of race and the rise of genetic determinism. First, in relation to research practices in genomics, ELSI scholars illuminate that how biomedical research has been racialized in terms of genomics, such that race has both been reconceived in genomic terms and has appeared as a biological essence (Fujimura et al., 2008). Particularly, their empirical studies reveal that labeling the activities of human populations and differences in genomic research plays a crucial role in making race a biological essence in terms of genomics (Fullwiley, 2007; Montoya, 2007; Fujimura and Rajagopalan, 2011; Panofsky and Bliss, 2017). Even though some genomic scientists consciously avoid the use of racial categories and adopt alternative concepts such as geographic or genetic ancestry, they rely on racial concepts when clustering genomic databases (Bolnick, 2008; Fujimura and Rajagopalan, 2011). In many cases, labeling activities in genomic research is strongly influenced by genomic scientists’ local understanding of groupings among human populations. As a result, local social categories of human groups—particularly racial categories in the U.S.—are reified as biological essences in the process of labeling practices in genomic research (Gannett, 2014). This biological reification of race in genomic research contributes to the rise of genetic determinism (Gannett, 2004).

Second, ELSI scholars reveal how media representations of genomic research on race strengthen genetic determinism (Nelkin and Lindee, 1995; Phelan et al., 2013). They show that news articles “distort” explanations about scientific outcomes in original press releases and play a vital role in “science hype” in relation to genomic research (Brechman et al., 2011; Caulfield and Condit, 2012). This problem of science communication and misrepresentation in the media is closely linked to journalistic norms (Nelkin, 1996). In contrast to scientists, journalists write for diverse readers that vary in their interest and knowledge levels. As a result, journalists often must simplify the implications of scientific outcomes. For instance, journalists will report that scientists discovered “a fat gene” instead of “a meaningful marker that may predispose an individual to obesity” (Nelkin, 1996, p. 1602). The media’s oversimplification of scientific results misrepresents modest genomic research on different human groups as a racial study implying genetic determinism and racist arguments (Phelan et al., 2013).

The problem of labeling practices in scientific research practice and the simplification of research results in media representations are similarly identified with the case of the geneticization of ethnicity and dietary habits in anti-doping genomic research. Indeed, in the case of ethnicity in *UGT2B17* research, the misuse of ethnicity can be understood in light of the problem of labeling practices. And in both cases (i.e., ethnicity and dietary habits) relating to *UGT2B17* studies and their media reports, simplification of scientific research created genetically deterministic and racist explanations on the association between sports doping and cultural identities and habits.

Meanwhile, one can identify different factors influencing the biological reification of race in anti-doping genomic research and other genomic sciences. Those differences are mainly due to the distinctive characteristics of anti-doping science. Anti-doping genomic research is clearly separated from academic genomic sciences in two ways: its regulatory and political aspects (**Table 1**). First, anti-doping science is a regulatory science that seeks to improve the regulation of doping in sports, rather than expand general knowledge of the natural world. As science scholar Sheila Jasanoff highlights, regulatory scientists are more focused on improving regulation methods than creating scientifically rigorous research outcomes (Jasanoff, 1990). This regulatory-oriented research approach allows anti-doping researchers to ignore research protocols and guidelines that scientists in academic-oriented disciplines have developed.

**Table 1:** Two characteristics of anti-doping science.

Anti-doping science	
**Regulatory nature**	**Political nature**

Regulatory science	Anti-doping policy
Regulatory-oriented	Unfairness-focused
Doping control of athletes	

Second, anti-doping science is directly influenced by anti-doping politics. Due to its regulatory nature, anti-doping science already takes a specific stance in anti-doping politics. Anti-doping scientists conduct their research with support from WADA’s anti-doping policy and share the political position as WADA: sports doping and doping-related works and traits are inherently unfair and should be eradicated (Kayser et al., 2007). This political involvement with WADA’s anti-doping policy influences the view of anti-doping scientists with respect to the useful aspects and implications of their research. The news media also share this political view of the unfairness of sports doping. This unfairness-focused concern of anti-doping research and its media representations often leads anti-doping scientists and the mass media to fail to recognize that they sometimes deliver ethically unjustified statements on specific groups.

Those two characteristics of anti-doping science are closely related to the influential power of athlete communities worldwide. Indeed, anti-doping scientists play a crucial role in exerting doping control over athletes. Their research outcomes are directly applied in regulatory practices in near-future international sports. For this reason, elite athletes and their coaches are strongly responsive to new information regarding state-of-the-art anti-doping research and are easily harmed by news reports on anti-doping studies, regardless of the correctness of media representations. In this respect, though its public is quite limited to athlete communities, anti-doping research and its media reports have a much greater impact on the public than other general academic sciences.

We can use this understanding of anti-doping science to examine the process of geneticization of ethnicity and dietary habits. First, the regulatory nature of anti-doping science makes labeling practices in anti-doping genomic research more problematic. Anti-doping scientists only concentrate on research outcomes that improve doping regulations, and are not concerned about the connotations of their scientific research. For example, they conflated “ethnicity” with “race” without serious consideration to the debate of grouping categories in human genomics research. Indeed, “ethnicity” has been a proposed alternative for replacing the term “race” in genomic research, yet anti-doping scientists did not consider this effort that had been occurring in the field of academic genomic sciences (Sankar and Cho, 2002). Panofsky and Bliss (2017) have shown that grouping categories within the human population remain unstandardized and ambiguous, yet most academic genomic scientists recognize the problem of using racial categories and thus try to avoid racial connotations. Anti-doping researchers failed to recognize the research protocols and guidelines developed by the academic genomics communities, and thus labeled their research populations using racial classifications.

Second, the political nature of anti-doping science, with its regulatory nature, influences anti-doping scientists’ reporting style and selective emphasis on research outcomes. In contrast to general academic sciences like genomic cancer research, a biased explanation on the research outcome is produced from the very early stages of the press release (Brechman et al., 2011). Using the moral judgment that sports doping is unfair and harmful, anti-doping scientists report the unfair aspects of some genetic variations and metabolic mechanisms in their research outcome. Harm reduction and protecting athlete health are two important rationales in anti-doping policies, yet those aspects are ignored when anti-doping scientists report their research outcomes (Kayser et al., 2007). Also, this regulatory nature strengthens this selective emphasis tendency when reporting their research outcomes. For anti-doping scientists, regulatory priority is emphasized to a greater degree than the sufficient accumulation of scientific evidence. For this reason, results that help the regulatory regime are selectively emphasized and the scientific contestation of their research outcome is often ignored. As I showed, WADA and anti-doping scientists focused on the misuse of green tea to avoid doping regulation, even though the supportive research was merely an *in vitro* study. In contrast, the negative *in vivo* study on the misuse of green tea was ignored by WADA officials and the wider anti-doping scientist community.

Third, in the mass media, socially problematic arguments are easily justified because of the political nature of anti-doping science. News media reports often use the moral judgment that sports doping is unfair and harmful when framing their articles, and thus reports anti-doping research outcomes by referencing possible “doping allegations” and future “scandals.” In order to prevent or criticize possible unfair situations in sports games, news reporters are allowed to make socially problematic arguments like Asian athletes are “genetically born to cheat” and have “a gene for doping with impunity.” By adopting this moral judgment on sports doping, news journalists do not adhere to the “objectivity” norm in journalism, that is, reporting and balancing conflict claims (Nelkin, 1996). This paper, for example, identified zero news articles that reported on the contradictory *in vivo* study questioning the ability of green tea misuse to avoid doping detection.

Most importantly, contrary to academic genomic sciences, anti-doping research and its media reports have a powerful impact on a specific lay group—athlete communities. As ELSI scholars of genomics and race reveal, most of the lay public is less influenced by the media coverage on genomics and race than some of critical racial scholars claimed. The lay public does not believe the media’s misrepresentation on academic genomic research and actively assesses the content of news articles using their complex view on race (Condit et al., 2004). In other words, the media representation of results in academic genomic sciences has a relatively limited influence to the public dimension. On the contrary, anti-doping research and its media representations immediately affect the lives of athletes. Regardless of the scientific concreteness of the claims, athletes and coaches worldwide can be enforced to change their cultural customs, like drinking green tea. Given their subordinate relationship within WADA’s anti-doping control system, athletes and coaches easily accept and internalize the genetic determinism and racist discourse generated by the media representations.

**FIGURE 1:**
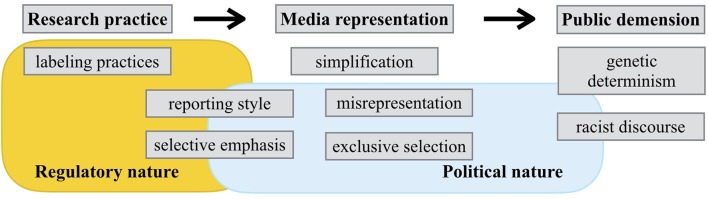
**The process of geneticization in anti-doping science**.

In sum, the process of geneticization in relation to anti-doping science shows both similarities and differences with cases of academic genomic sciences that ELSI scholars in genomics and race have studied (**Figure 1**). Cases of anti-doping genomic research share similar problems with other genomic studies regarding labeling practices in research practice and outcomes simplification in media representation. At the same time, due to the regulatory and political natures of anti-doping science, anti-doping genomic research and its media reports more easily facilitate genetic determinism and racist discourse in the public dimension. By understanding such distinctive characteristics of anti-doping science, ELSI scholars can begin to apply a more critical analysis on the social impact of this science in the context of genomics and human enhancement.

## Conclusion

In this article, I tried to show the social impact of the genomic knowledge that anti-doping scientists have produced and that the news media has propagated. I pointed out the geneticization of cultural objects such as ethnic identities and dietary habits by anti-doping science, and proposed the need for increased attention to this science by ELSI scholars who explore human enhancement and genomic science. Furthermore, I engage with the ELSI scholarship on genomics and race to suggest that an understanding of the regulatory and political natures of anti-doping science is a possible starting point for including anti-doping science in ELSI programs in human enhancement and genomics.

The ethical debates on the implications of gene doping and other potential doping technologies remain important. At the same time, however, one should recognize the fact that the realization of doping technologies is still in the future whereas the social impact of anti-doping science is in the present. For this reason, ELSI scholars should include this regulatory science in their analysis if they want to thoroughly investigate which ethical and societal problems are raised with respect to the topic of sports enhancements and genomic science.

## Author Contributions

The author confirms being the sole contributor of this work and approved it for publication.

## Conflict of Interest Statement

The author declares that the research was conducted in the absence of any commercial or financial relationships that could be construed as a potential conflict of interest.

## Footnotes

1 For an extensive review of the ethical problems of human enhancement, see Allhoff et al. (2011).
